# Evaluation of employee occupational stress by estimating the loss of human capital in Japan

**DOI:** 10.1186/s12889-022-12751-7

**Published:** 2022-03-01

**Authors:** Xiangdan Piao, Shunsuke Managi

**Affiliations:** 1 Faculty of Humanities and Social Science, Iwate University, 3-18-34 Ueda, Morioka, Iwate 020-8550 Japan; 2 Urban Institute, Kyushu University, 744 Motooka Nishi-ku, Fukuoka, 819-0395 Japan; 3 Urban Institute & Department of Civil Engineering, Kyushu University, 744 Motooka Nishi-ku, Fukuoka, 819-0395 Japan

**Keywords:** Stress, Employee, Japan, Human capital, Psychological well-being

## Abstract

**Background:**

Human capital is thought to be a crucial factor that drives economic growth. This study aims to understand the evaluation of the loss of human capital caused by employees’ occupational stress.

**Methods:**

In total, 1,021,178 observations for employee occupational stress were collected from 390 companies from 2017 to 2019 in Japan. The original cross-sectional survey contains 11,167 employees with occupational stress and their socioeconomic information in 2015. The relationship between stress and annual income is estimated with polynomial regression, and accumulated human capital loss is estimated. Matching approaches are applied for corporate human capital loss.

**Results:**

The negative association between annual income and employee stress is derived, which indicates that the worse the employees’ stress is, the greater the human capital losses. Importantly, we confirmed that most employees have human capital loss, and on average, for male employees aged 25, the accumulated human capital loss will reach approximately $0.6 million USD by retirement.

**Conclusion:**

For corporations, human capital loss is highly correlated with the number of employees, suggesting that reducing the occupational stress of employees can lead to greater corporate performance.

**Supplementary Information:**

The online version contains supplementary material available at 10.1186/s12889-022-12751-7.

## Background

Human capital is thought to be the most important driving factor in economic growth [1, 2]. Greater human capital growth is associated with sustainable economic development and sustainable societies [3, 4]. A sustainable and inclusive development society in 2030 is suggested by 17 clear multidimensional goals for human well-being prescribed by the United Nations [5]. For example, good health and well-being (goal 3), decent work and economic growth (goal 8), and responsible consumption and production (goal 12) are expected to improve population well-being and sustain development.

From the perspective of ecological economics sustainability, inclusive wealth, which is an aggregate of natural capital, produced capital, and human capital that measures society’s wealth, is considered an appropriate statistic to indicate whether society is advancing toward sustainability [3, 6–8]. Human capital is one of the core factors within and shares the largest proportion of the inclusive wealth index; empirical studies have found that it shares 65% of world inclusive wealth. Similarly, in Japan, human capital has the main share (60%-72%) of total aggregated wealth [8–11].

Human capital is crucial for the economic growth and sustainability of society [2, 12–17]. Human capital is a summation of the individual’s knowledge and skills [15, 16]. In pioneering wealth outcome studies, human capital is developed with the earning function and factors of year of schooling and years of experience to present a person’s knowledge and skills [15, 17]. In subsequent studies, human capital is developed in terms of income and years of schooling [6]. Investments in human capital are theoretical, and empirical studies are widely established [2, 10–20]. The established factors that improve human capital generally include education, skills, job training, and health [9, 16, 18–20].

In medical institutions, improving the psychological well-being of employees is treated as a more important issue, and improvement of occupational stress among employees by speeding up the workplace is proposed. On the other hand, companies have a strong tendency to pursue profits for sustainable corporate management. Labor management for the well-being of employees is one of the themes that companies focus on. Interprofessional collaboration from the perspectives of human capital theory and occupational stress will present the value of human capital loss associated with employee occupational stress, and evidence based on new insights may help explain the impact of occupational stress on policy makers and managers.

On the other hand, theoretically, for psychological risk factors, the job-demand-resource model or job-strain model has been widely documented [20–46]. The job strain model, developed by Schnall et al. [21], describes a theoretical concept for workplace characteristics and employee occupational stress. The job-strain model and job-demand-support model consider the worse employee occupational stress associated with greater workload demand and lower control of the workload process. Job demand contains the quantity and quality of the workload, and job control is the ability to control the work process or skill utilization. Aside from the job strain model, the job-demand-resource model argues that worse employee occupational stress is associated with high job demand and low surround support [22, 23]. Low levels of resources contain poor surrounding support, e.g., family, boss or colleagues.

In accordance with the above concepts and models of stress, a large body of empirical evidence has accumulated in recent years. Along with the models, work environment characteristics such as surround support, balanced effort and reward, work life balance and workplace violence appear to have an impact on employee occupational stress [22–46].

However, the issue needs to be examined. For example, the World Health Organization [46] argues that global psychological well-being and mental illness will lead to disease burden by 2030. Studies have found a negative relationship between stress in the work environment and employee performance [47–50]. The absence of evaluation of performance loss by stress in general employees might underestimate the social loss from worsening psychological well-being. When human capital plays a substantial role in the sustainability of society, the evaluation of human capital loss by employee job stress might build on the literature for improving human capital and, therefore, provide insightful evidence to illustrate how employee psychological well-being contributes to a sustainable society. We are motivated by the idea that a decent workplace is associated with better employee outcomes, and subsequently, the sustainability of society [4, 50]. Thus, we conducted this analysis using 1 million employee records of occupational stress.

This study builds on the literature in the following ways. We first investigate the relationship between human capital loss and employee occupational stress, which mainly focuses on male employees, using an original survey conducted in 2015. Second, the instrumental variable strategy is employed to address the potential reverse effects that low-paid employees might be employed in stressful workplaces. Third, corporate human capital loss regarding male employee occupational stress is illustrated using a matching approach with large-scale employee occupational stress collected from 2017 to 2019. The results of this study are expected to provide new insights for policy makers and corporate managers by first valuing human capital loss when presenting occupational stress.

The outline of this study is as follows: Sect. 2 presents the data and variable settings. Section 3 presents the methodology, and Sect. 4 summarizes the empirical results. Finally, Sect. 5 presents the discussion.

### Data

Two kinds of datasets, (1) large-scale employee job stress data (panel data) and (2) data from a cross-sectional original internet survey, are used in this study to show the loss of human capital by stress among male employees in Japan. We used two datasets because the large-scale employee longitudinal data do not have employee income information, which is necessary in human capital evaluation.

Referring to the Stress Check Program under the guidance of the Ministry of Health, Labor and Welfare (MHLW), the Japanese government requires companies with more than 50 employees to participate in the government-directed occupational stress check program for employees [51]. Companies with more than 50 employees are required to participate in a government-directed occupational stress screening program for employees. Often, at the request of the government, these companies commission a third-party psychological welfare company to check their employees' occupational stress levels. PEACEMIND Inc. is located in Tokyo, the capital of Japan. The target population is all employees when the stress check system was introduced. The questionnaire prepared by the Ministry of Health, Labor and Welfare asked respondents questions about psychological stress at work and the working environment through online and offline questionnaires.

**Fig. 1:**
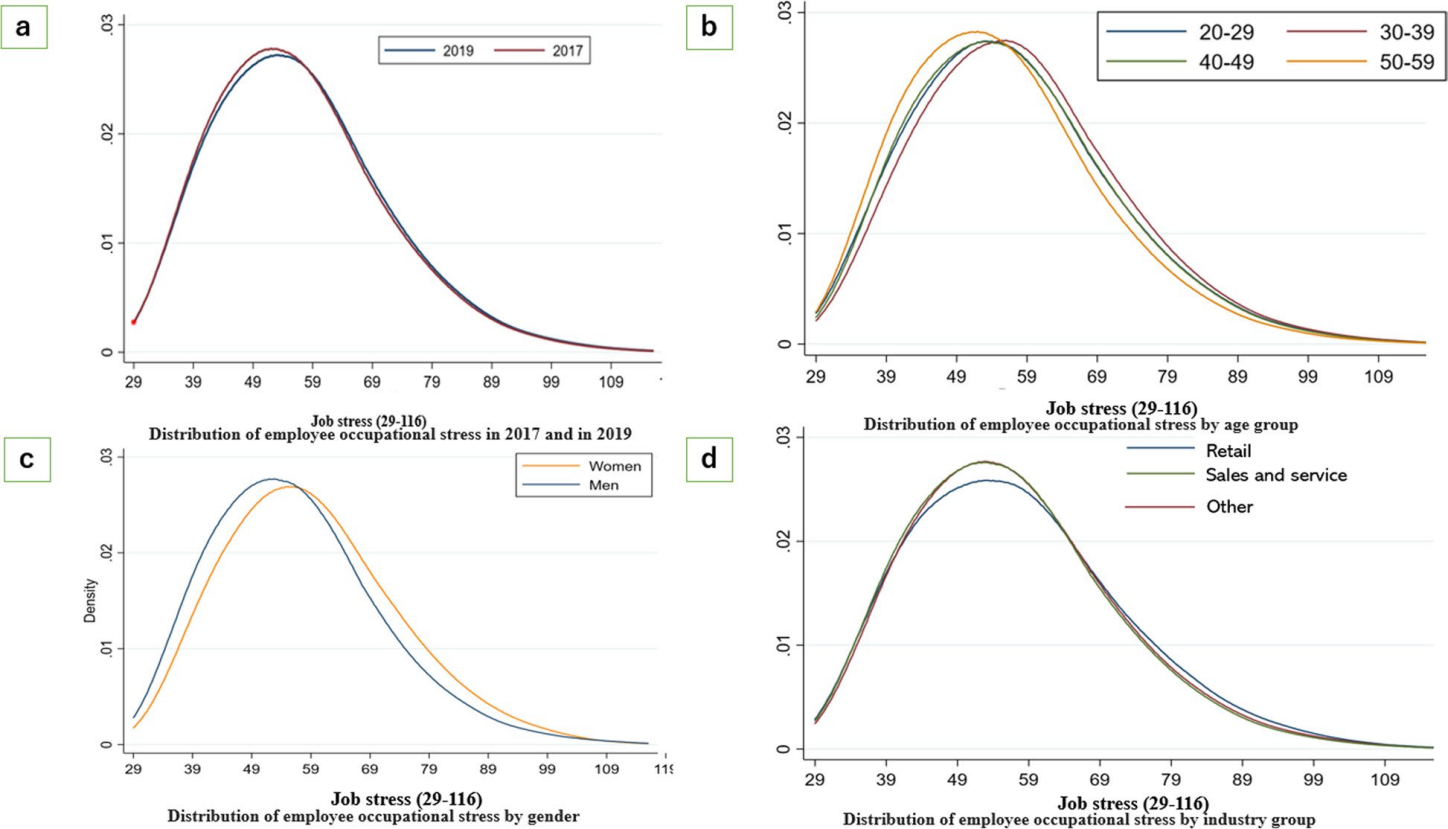
Distribution of employees’ job stress by group. Data sources: Large-scale employee longitudinal data

When the companies introduced the stress check program based on the guidelines of the Ministry of Health, Labor, and Welfare [51], the targeted respondents were employees belonging to the companies. The large-scale employee longitudinal data have more than 1 million observations collected from 2017 to 2019 in Japan. The study design was approved by the appropriate legal and ethics review board of PEACEMIND Inc. The data were provided with informed consent from participants, according to legal and ethical guidelines. The data in this study do not target personal health information, and personal information is nonidentifiable. All the methods were performed in accordance with ethical guidelines and approved by the ethical committee of PEACEMIND Inc. Targeted respondents were informed to participate in the stress survey, and high-qualified longitudinal data with low missing values (e.g., 0.48% in 2018) were collected. In total, 1,021,178 observations of 363,993 employees were successfully collected from 390 companies. Low missing values in the occupational stress program, i.e., 5,216 observations, were recorded over three years. Finally, 1,015,962 observations are used in the analysis. As several corporations did not collect the employee age information, after deleting the observations of women employee, the 657,185 observations are utilized in the lifetime human capital estimation analysis (see Fig. 1).

We conducted an original cross-sectional internet survey through a third-party company (Nikkei Research Company) from July to August 2015. Nikkei Research has provided some highly reliable internet survey services for the past few decades with a comprehensive panel to ensure that the sample fits a population’s gender and age distribution. We randomly selected those who met the requirements from the registered panel to distribute the survey. Nikkei research has an extensive panel to allow the selected random assignment targeted respondents to match the population gender and age distribution of Japan.^1^ The study design was approved by the appropriate legal and ethics review board of Kyushu University. The data were collected with informed consent from participants, according to legal and ethical guidelines. All the methods proceeded in accordance with ethical guidelines and were approved by the ethical committee of Kyushu University. We employed an extensive panel to allow the targeted respondents to match the general population distribution of Japan.^2^

The Pew Research Center [52] points out that internet-based surveys tend to select well-educated respondents with high household incomes. In total, 11,167 respondents from prefectures were collected. After deleting the observations of nonworking individuals as well as observations of individuals aged less than 20 and older than 65 years, there were 2,300 working women and 3,738 working men in our analysis (see Table 1). Please see a detailed description of the data for this survey in Chapman et al. [53]. The working individual’s GHQ-12, household income status, and detailed demographic information were collected.

**Table 1:** Description of original internet survey and employee occupational stress data in Japan

Data Original Internet survey	Data Employee occupational stress	
Survey participation (*n* = 11,167)	Survey participation (number of participation = 390)	Numbers of observation for occupational stress from 2017 to 2019. *n* = 1021,178
Participations ageless than 20 and more than 65 and missing value in key variables (*n* = 5129); women employees (*n* = 2,300)		Missing value in occupational stress 5,216 observations in occupational stress, Observations in women employees = 363,993
Analysis: Relationship between human capital and employee stress. Male employees (*n* = 3738)	Analysis: Corporates human capital loss by employee occupational stress (*n* = 390)	Employee capital loss by occupational stress (*n* = 1,015,962) Lifetime human capital loss by occupational stress (*n* = 657,185)

### Variable setting

#### Job-stress-29

The Job-stress-29 is part of the Brief Job Stress Questionnaire based on the guidelines of the Japanese Ministry of Health, Labor, and Welfare [51]. The Job-stress-29 has 29 items to measure mental health status as follows: The following are questions on how you felt during the past month. Please choose the most applicable answer. 1) I have been very active; 2) I have been full of energy; 3) I have been lively; 4) I have felt angry; 5) I have been inwardly annoyed or aggravated; 6) I have felt irritable; 7) I have felt extremely tired; 8) I have felt exhausted; 9) I have felt weary or listless; 10) I have felt tense; 11) I have felt worried or insecure; 12) I have felt restless; 13) I have been depressed; 14) I have thought that doing anything was a hassle; 15) I have been unable to concentrate; 16) I have felt gloomy; 17) I have been unable to handle work; 18) I have felt sad; 19) I have felt dizzy; 20) I have experienced joint pains; 21) I have experienced headaches; 22) I have had a stiff neck and / or shoulders; 23) I have had lower back pain; 24) I have had eyestrain; 25) I have experienced heart palpitations or shortness of breath; 26) I have experienced stomach and / or intestine problems; 27) I have lost my appetite; 28) I have experienced diarrhea and / or constipation; 29) I haven’t been able to sleep well. Answer choices were as follows: Almost never = 1; Sometimes = 2; Often = 3; Almost always = 4. Therefore, job-stress-29 is the nonweighted summation of the 29 items, ranging from 29 to 116, with a greater value indicating worse mental health. The score was reversed for 1), 2), and 3).

The Japanese Ministry of Health, Labor and Welfare identifies highly stressed employees using a threshold score derived from a job-stress-29 score as individuals at high risk for mental health disorders. One of the standards defined employees as highly stressed with a threshold score of 77, according to the Japanese government; a large proportion of the employees who scored less than 77 also have a potential risk of becoming high-risk employees. Therefore, job-stress-29 can be adopted as an indicator of employees’ mental health status [54, 55].

#### GHQ-12

The generalized health question (GHQ-12) has 12 items regarding mental health and was included in our original internet survey. The questions are as follows: Please select one option that is the most applicable. Have you recently (1) been able to concentrate on whatever you are doing? (2) lost much sleep over worry? (3) felt that you are playing a useful part in things? (4) felt capable of making decisions about things? (5) felt constantly under strain? (6) felt you could not overcome your difficulties? (7) been able to enjoy your normal day-to-day activities? (8) been able to face up to your problems? (9) been feeling unhappy and depressed? (10) been losing confidence in yourself? (11) been thinking of yourself as a worthless person? (12) been feeling reasonably happy, all things considered? Responses were measure on a four-point scale: Not at all = 4; No more than usual = 3; Rather more than usual = 2; and Much more than usual = 1. In this study, we reversed the four items as Not at all = 1; No more than usual = 2; Rather more than usual = 3; and Much more than usual = 4, and the GHQ-12 score (ranged 12–48) was a nonweighted summation of the above 12 items, which indicates that the greater the score is, the worse the mental health status.

#### Other variables

The original internet survey includes age dummies (20–29, 30–39, 40–49, 50–59, and 60–65 years), occupation categories (full-time employee, part-time employee, and other) and employee annual income.

### Methodology

The local polynomial regression model, which is a generalized local mean smoothing model, was adopted to investigate the nonlinear association between employee stress and an individual’s annual income. Both local polynomial regression and Robinson double residual estimation are appropriate when examining the relationship between stress and employee income [56–58]. The results derived from the instrument strategy are used as a robustness check. The local polynomial regression model is as follows:

1 $${S}_{i}=f({x}_{i})+{\varepsilon }_{i}$$

where ${S}_{i}$ denotes employee $i$’s annual income, and ${x}_{i}$ is the stress score (GHQ-12). The function $f(.)$ indicates the nonlinear function of the stress score (GHQ-12) $x$. The error term is ${\varepsilon }_{i}$.

It is possible that low-paid employees might be employed in more stressful working environments, which is thought to be a reverse causal relationship. To address the problem, we employed an instrumental variable strategy, and the two instrumental variables (Z) were (1) the weather on the survey day recorded by respondents and (2) the survey completion time for each respondent to assess individuals’ psychological well-being [57]. The weather status on the survey date (e.g., sunny) is known to be correlated with mental well-being ($x$) but is not correlated with the individual’s annual income. Again, ${x}_{i}$ is the stress score (GHQ-12), whereas $\widehat{{x}_{i}}$ is the imputed value according to the estimation of the first-stage regression (Eq. 2).

2 $${x}_{i}={\theta }_{0}+{{Z}_{i}\theta }_{1}+{Y}_{i}{\theta }_{2}+{\varepsilon }_{i}$$

3 $${S}_{i}={\alpha }_{0}+{\alpha }_{1}\widehat{{x}_{i}}+{Y}_{i}\beta +{\varepsilon }_{i}$$

${S}_{i}$ presents the individual $i$’s income. $Y$ denotes the independent variables of socioeconomic and demographic variables. ${\theta }_{0}$, ${\theta }_{1}$, ${\theta }_{2}$, ${\alpha }_{0}$, ${\alpha }_{1}$ and $\beta$ are estimated parameters. The error term is ${\varepsilon }_{i}$. The parameter ${\alpha }_{1}$ presents the impact of employee stress on the individual’s income.

The major concern is the estimation of one unit of human capital loss by job stress by stress level $j$, which can be derived from Eq. (4), based on the microapproach. In the development theoretical framework of human capital, a pioneer of wealth output who developed human capital as a function of income with years of education and years of experience as elements presented knowledge and skills [15–17], and subsequent studies have used income and years of attendance years of schooling [6]. In line with the theoretical framework of human capital and development in human capital loss in employee occupational stress, income is thought to be an appropriate measurement of occupational stress-induced productivity loss in terms of the accumulation of skills and knowledge.

4 $$H_j={\textstyle\sum_{t=1}^T}L_j\left(\frac{1+a}{1+b}\right)^t$$

One unit of human capital loss denotes ${H}$, which is the accumulated amount of income from the employee’s current age to retirement age $T$. The amount of income loss by stress level $j$ in each year is denoted as ${L}_{j}$, which is the difference between the stress-free status annual income and employee income when he or she feels stress at stress level $j$ estimated from Eq. (1). $a$ is the income growth in each year capturing the accumulation of human capital between stress-free status and stressful status, which is assumed to be 2%. Similarly, the year interest rate $b$ followed the Bank of Japan and was 0.004%.^3^

Aside from the above microapproach, following Arrow et al. [6], the macroapproach of estimating human capital is also conducted. The one-unit human capital loss by stress level $j$ is derived from Eq. (5). The stress level $j$ has an 88 average score ranging from 29 to 116, which indicates that the greater the number of stress scores is, the worse the psychological stress.

5 $$H_j={\textstyle\sum_{t=1}^T}L_j\left(\frac1{1+\delta}\right)^t{\ast\exp}^{\rho E}$$

The returns of the years of schooling $\rho$ are 8.5%, and the average year of schooling for Japanese males $E$ is 15.47. The discount rate $\delta$ is 5%. $P$ is the number of male employees in companies, where ${P}_{j}$ is the number of employees in a company at the stress level$j$. The amount of income loss by stress in each year is denoted as$L$. The shadow price of one unit of human capital by stress at level $j$ is ${p}_{j}={\sum_{t=1}^{T}{L}_{j}{\left(\frac{1}{1+\delta }\right)}^{t}}$. Accordingly, one-unit human capital loss ${H}_{j}$ at the stress level $j$ becomes${p}_{j}{*\mathrm{exp}}^{\rho E}$. The human capital loss by stress in company $M$ is the summation of one-unit human capital loss by each stress level,$j$, and the number of employees at each stress level ${P}_{j}$ is shown in Eq. (6).

6 $$M={\textstyle\sum_{j=1}^{88}}p_j\ast P_j\exp^{\rho E}$$

The relationship between individuals’ annual income and his/her occupational stress is displayed using the local polynomial model presented in Eq. (1). Based on the results derived from the above regression, the income loss (${L}_{j}$) compared to the individuals’ who are stress free is calculated after regression. The expected results are that the worst employee occupational stress will be associated with higher annual income loss. The human capital loss derived from accumulated annual income until retirement is illustrated in Eq. (4). The corporate human capital loss from employee occupational stress is the unweighted summation of the income loss due to stress of each employee. It is derived from the employee responses to prompts about occupational stress on the survey, and the annual income loss is derived from Eq. (1) using the exact math approaches.

## Results

### Basic statistics

Fig. 1 displays the kernel distribution with bandwidth 3 of employees’ job stress by various groups using the large-scale employee longitudinal data. The job-stress-29 score ranged from 29 to 116, which indicates that the greater the number, the worse the psychological distress. It is notable that in each subgroup divided by age, gender, year, or sector, the pattern of employee psychological stress distribution is similar. This indicates that aside from the high-stress threshold score of 77 provided by the Japanese government, a large proportion of the employees who scored less than 77 have a potential risk of becoming high-risk employees. Fig. 1 (a) illustrates the employee’s stress distribution for the years 2017 and 2019, which shows that the employee in 2019, on average, was slightly worse by 0.42 points. This is consistent with the global trends of increasing burden of mental illness, according to the World Health Organization [18]. Fig. 1(b) illustrates that employees aged 30–39 have a higher stress level than other age groups. Similarly, when compared to men, women tend to have worse psychological stress (Fig. 1(c)). Focusing on the various industry sectors, we found that employees from the retail sector have higher stress than employees from other sectors (Fig. 1(d)). With regard to the industry in detail, the psychological job stress distributions are close to the summarized employee stress distribution displayed in Fig. 1(d).

**Fig. 2:**
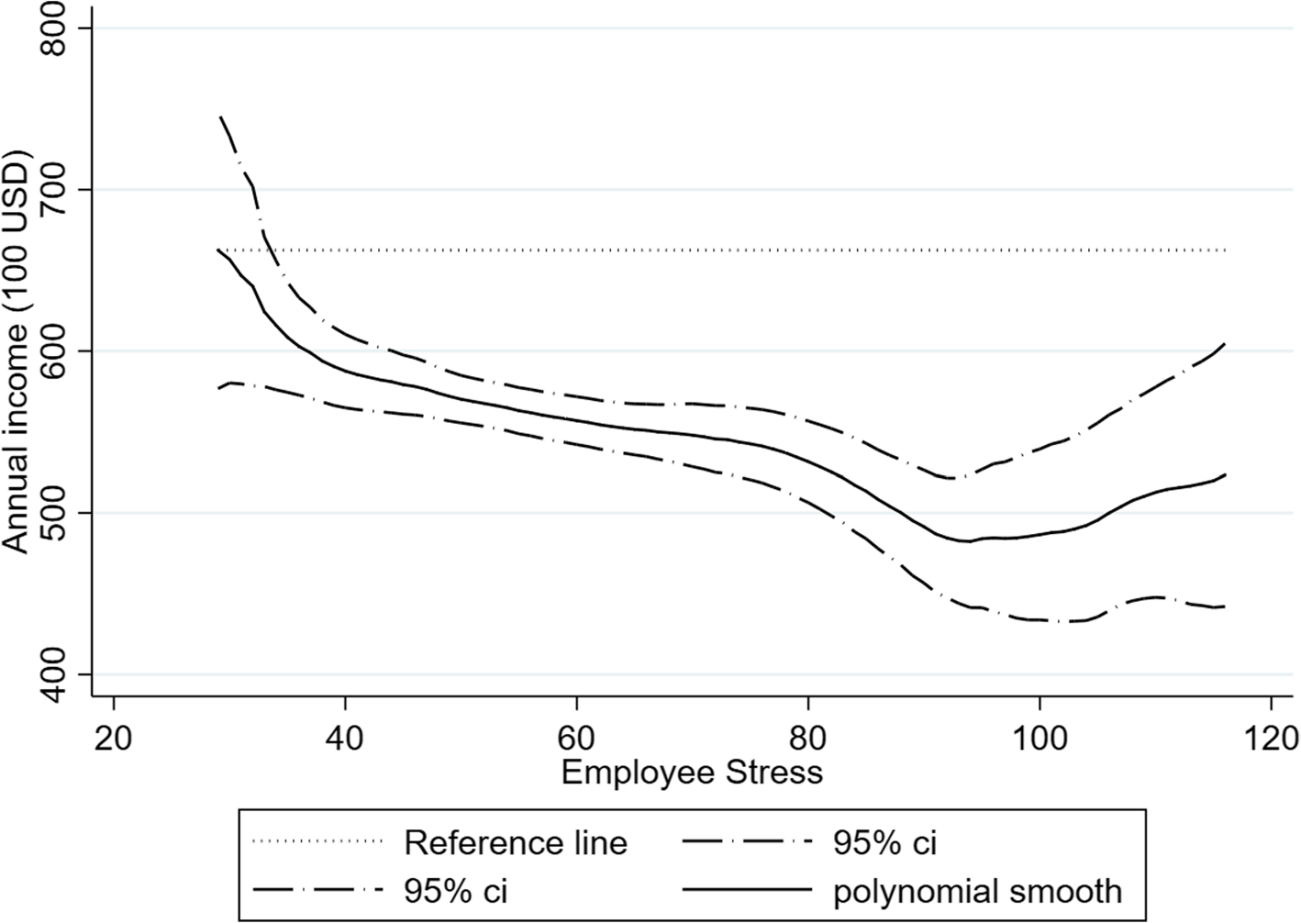
Association between employee stress and annual individual income

Fig. 2 illustrates the estimation results of the local polynomial regression model regarding the nonlinear relationship between income and stress using the original 2015 internet survey in Eq. (1). The dependent variable is annual income, and the independent variable is male employee stress. Employee stress is measured by GHQ-12 rearranged to 29 to 116, where a greater number indicates a worse stress level. The reference line represents the stress-free annual income, which is the annual income equal to $66,250 USD, whereas the yearly human capital loss at the $$j$$ th stress level, $${L}_{j}$$, is derived by differencing the reference line and polynomial smooth line. In this study, the exchange rate of USD and Japanese yen is 1 USD, which equals 100 Japanese yen [59]. The dashed-dotted line indicates the 95% confidence interval (CI). The black line is the zero-degree polynomial smooth with kernel function using the rule-of-thumb (ROT) bandwidth estimator. Both robust results were obtained from a local polynomial regression with a cubic polynomial smooth (Eq. 1) [60, 61].

### Relationship between employee stress and annual income

#### Data sources: original internet survey

Table 2 displays the results from the instrumental variable strategy to address the reverse effect that low-paying employees might be employed in more stressful workplaces using the weather on survey day and survey completion time. The dependent variable is an individual’s annual income, and the independent variable of interest is employee stress. The value of the first-stage F test is 10.54 greater than 10, which suggests that the instrumental variables are not weak instruments. Similarly, the Sargan score was 2.66, with a p value of 0.44, indicating that the two instrumental variables were appropriate.

**Table 2:** Relationship between psychological well-being and individual’s annual income based on two-stage least square

	(1)
VARIABLES	ln(individual income)
Ln(GHQ-12 score)	-0.671*
	(0.400)
Control variables	Yes
R-square	0.1624
Sargan (score)	2.66
*p* value	0.44
First-stage F test	10.54
*p* value	< 0.0001
Observation	3,738

The two instrumental variables are (1) the weather on the survey day recorded by respondents and (2) survey completion time for each respondent. Control variables: education, age, number of children, number of family members, occupation, house status

Importantly, the coefficients of an individual’s psychological well-being (GHQ-12 score) are negative and statistically significant. The results suggest that when an employee’s psychological well-being worsens by 1%, his or her annual income decreases by 0.671% when the reverse effects are addressed and demographic and socioeconomic characteristics are controlled. The negative impact of employee stress on human capital outcomes occurs through a decrease in annual income.

The results are summarized as follows: The slope of the black line turns downward as the employee stress score increases. This indicates that a negative association between employee stress and annual income is found in Japan, which shows that worse employee stress is associated with lower annual income. The relationship between stress and income is nonlinear, and the sharp income decrease with increasing stress is between stress scores 29–38 and stress scores 76–93. In contrast, a slow income decrease was confirmed between the stress level scores of 39 and 75. This might suggest that although the increasing stress score presents psychological distress, it does not uniformly decrease employee performance. However, an employee stress status greater than 76 seems to be a threshold that should trigger concern. This result is consistent with the psychological distress of employees defined by the Japanese government.

The results highlight that the stress-free stress score is 29, and annual income records the highest income level. In contrast, the stress score at 93 records the lowest income level, which is approximately $48,208 USD. The income loss at stress score 93 is $18,000 USD each year, which indicates a large amount of human capital loss owing to employee stress status. The annual human capital loss by different stress statuses ranges from $0 to $18,000 USD for an average of $11,920 USD. In sum, the results suggest that when employee stress status is improved by introducing government policy, a significant increase in performance is realized.

#### Employee and corporate human capital loss due to psychological stress

**Fig. 3:**
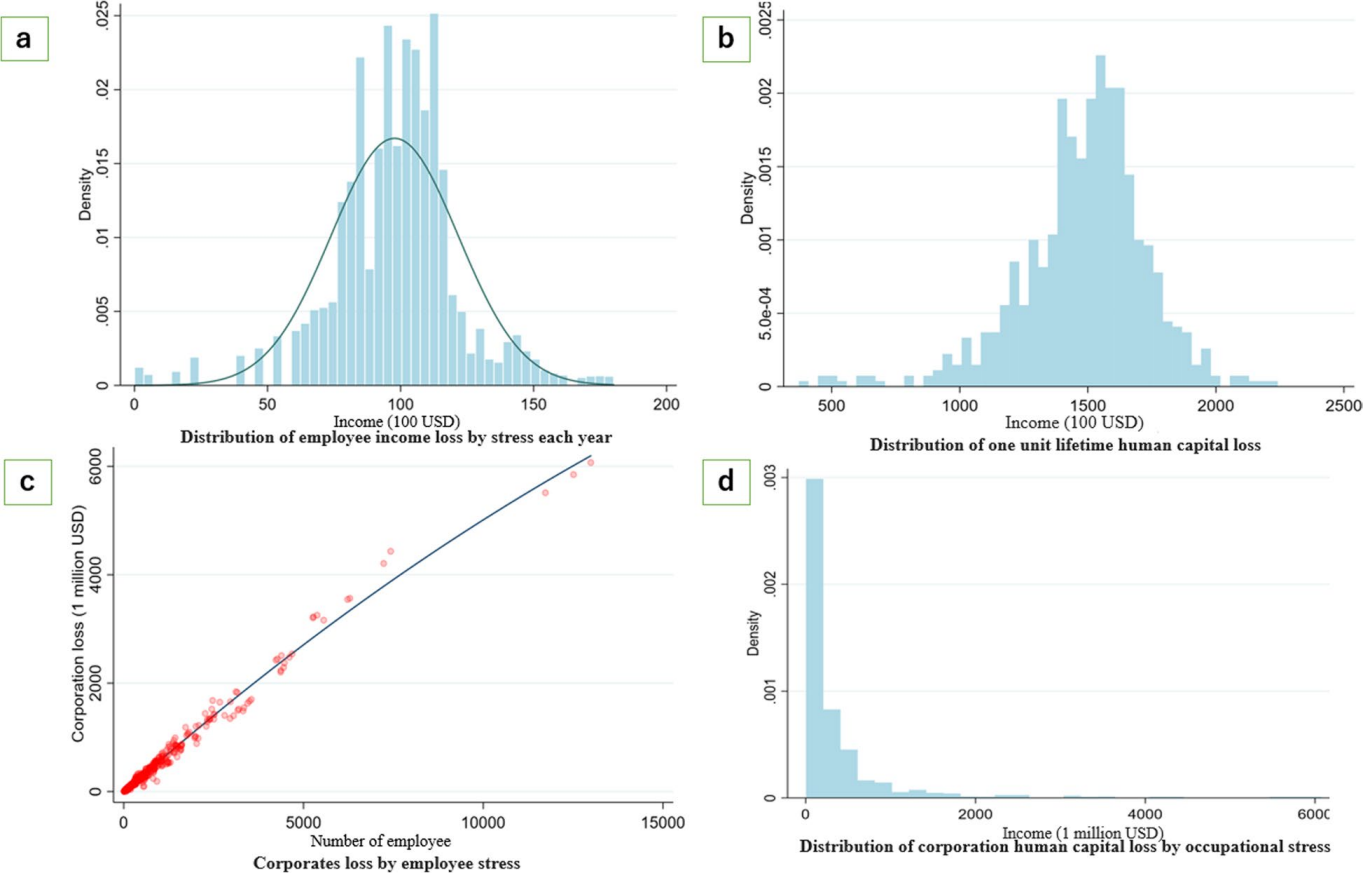
Employee and corporate human capital loss by stress in Japan

Fig. 3 shows the male employee and corporate monetary valuated human capital loss by psychological stress using large-scale employee longitudinal data. The employee annual income loss by stress is calculated by taking the difference between the stress-free status annual income and annual income corresponding to psychological stress based on the results from Eq. (1). The employee self-rated psychological stress ranges from 29 to 116, in which the corresponding annual income loss by stress is between $0 and $18,000 USD. By matching^4^ income loss to each employee in employee longitudinal data using each individual’s psychological stress, the distribution of income loss by stress each year can be displayed (Fig. 3 (a)). The monetary valuated human capital losses are obtained from Eq. (1). The annual income loss by stress, employee psychological stress score (Job-stress-29 score), and employee age and the distribution are shown in Fig. 3 (b). The 390 companies’ corporate loss is the sum of the human capital loss owing to the stress of all male employees as shown in Fig. 3 (c) and 3(d).

The results are summarized as follows: The distribution of income loss by stress shows that most employees lose more than $5,000 USD income each year, which shows that human capital loss owing to psychological distress is common among employees from various companies or industries (Fig. 3 (a)). The reality that human capital loss exists for most employees rather than a very small specific proportion of employees might attract policy makers’ attention. Improving employee stress levels may increase the employees’ performance. Intuitively, employee income loss by stress each year, on average, is approximately $10,000 USD (Fig. 3 (a)). Fig. 3 (b) distributes the lifetime human capital loss based on Eq. (5). Similarly, a large amount of human capital loss is observed universally among the employees, which is close to Fig. 3 (a). For example, the average employee job-stress score is 57 among employees aged 20–29 years. Based on the simulation, when the employee stress-level condition is not improved, the lifetime human capital loss accumulated to 65 years old will reach $600,000 USD for each employee.

When we focus on corporate human capital loss due to employee stress, corporate loss is highly correlated with the number of employees (Fig. 3 (c)). This is because, as shown in Fig. 3 (a), most employees have human capital loss by stress, and the loss ranges from $5,000 to $18,000 USD. Therefore, the more employees the companies have, the greater the loss might be. It is worth noting that large companies and nonretail sectors show better performance in terms of human capital loss owing to stress. Among the 390 companies included in our datasets, the median corporate loss is $101 million USD, which shows potential for improvement of the impact of employee psychological stress on company finances.

As the main results were derived from large-scale employee longitudinal data provided by a third-party company, there may be a problem in sample selection. To check the problem of sample selection, we conducted a robustness check using another sample provided by a fourth-party company having a sample size of 350,000 collected from over 100 companies and public institutions in Japan. Based on the methodology introduced in the methodology section, we confirmed the robust results based on employee stress.

## Discussion

This study first illustrated the stress-related human capital loss to fill the knowledge gap in the relationship between human capital and employees’ performance loss caused by stress in Japan. The study used large-scale longitudinal data from 390 companies based on data from the Japanese Ministry of Health, Labor and Welfare [51] collected by a third-party company from 2017 to 2019 and an original internet survey conducted in 2015 through a third-party company. Polynomial regression was used to investigate the association between monetary-evaluated performance loss and employees’ stress levels, and the instrument variable strategy was used as a robustness check. Moreover, the human capital loss due to stress was calculated at the individual and corporate levels.

First, employees’ psychological well-being (stress) was worse in 2019 than in 2017, which is in line with the expectation of the world health organization [46]. We found that employees aged 30–39, women, and retail sector employees have worse psychological stress levels than other corresponding groups. Second, the negative association between income and psychological stress among male employees shows that the annual human capital loss due to different stress statuses ranges from $0 to $18,000 USD. Importantly, human capital loss exists in most employees who lose the income of more than $5,000 USD each year, and human capital loss owing to psychological stress is common among various companies or industries. Third, the corporate human capital loss is highly correlated with the number of employees, which suggests that improving employees’ psychosocial well-being could contribute significantly to corporate outcomes. The median outcomes are expected to be $101 million USD. The results are consistent with the previous occupational stress literature based on the job-strain model or job-demand-resource model indirectly [20–46]. Both highlight improvement of the workplace environment for employees positively associated with the corporates’ outcomes.

The policy implications based on the results of this study are as follows. The Japanese government Ministry of Health, Labor and Welfare [51] introduced a simple employee stress check program to understand employee stress status. Given that high-stress employees are more likely to attract policy makers’ attention, based on our results, the policy improves overall employees’ psychological well-being and may produce large corporate outcomes. To improve the working environment quality, the Karasek job-demand-control model is recommended [21–40], as it shows that a better working environment is characterized by employee control of work procedures, use of skills in the workplace, and appropriate work demand.

This study aims to illustrate the relationship between employee human capital and occupational stress in Japan. However, the limitation of this study is acknowledged as follows. First, potential bias might be caused by the sample collection method. The large-scaled occupational stress is collected from the employees’ psychological well-being is more likely close to Tokyo from 2017 to 2019, which might not be a representative sample to the population. Though we used another employee occupational stress sample focus on the south area of Japan and confirmed the robustness. The major results are still a sample selection bias exist; further in-depth study should be based on the relevant datasets. Second, for the issue of an endogenous problem on the employee stress, we confirmed the robustness results based on instrument variable strategy using the weather at survey day record by respondents and survey completion time for each respondent are employed for individuals’ psychological well-being as the instrument variables. Combining with the original cross-sectional survey characters, the instrumental variable strategy is adopted, in future work, the panel data with a more relevant analysis methodology might be relevant to address the endogenous problem.

## Supplementary Information

**Additional file 1.**

## Data Availability

The data are publicly unavailable; however, the data are accessible when PEACEMIND Inc. provides data access permission, and other deidentified data for the current study are available from the corresponding author on reasonable request.

## Abbreviations

GHQ-12: The 12-Item Generalized health question
JPSC: Japanese Panel Survey of Consumers
ROT: Rule-of-thumb

## Footnotes

1 The respondent rate is not recorded.

2 The respondent rate is not recorded.

3 We followed the time deposit interest-rate guided by the Bank of Japan [58].

4 Due to large-scale employee longitudinal data on employee stress, this estimate does not include income information. We used two datasets.

## References

[1] Cinnirella F, Streb J (2017). The role of human capital and innovation in economic development: evidence from post-Malthusian Prussia. J Econ Growth.

[2] Gennaioli N, La Porta R, Lopez-de-Silanes F, Shleifer A (2013). Human capital and regional development. Q J Econ.

[3] Managi S, Kumar P (2018). Inclusive wealth report.

[4] Angrist N, Djankov S, Goldberg PK, Patrinos HA (2021). Measuring human capital using global learning data. Nature.

[5] United Nations. Sustainable development goals. 2015. https://www.un.org/sustainabledevelopment/sustainable-development-goals/. Accessed 20 July 2020.

[6] Arrow K, Dasgupta P, Goulder L, Mumford K, Oleson K (2012). Sustainability and the measurement of wealth. Environ Dev Econ.

[7] Polasky S, Kling CL, Levin SA (2019). Role of economics in analyzing the environment and sustainable development. PNAS.

[8] Yamaguchi R, Sato M, Ueta K (2016). Measuring regional wealth and assessing sustainable development: An application to a disaster-torn region in Japan. Soc Indic Res.

[9] Zhang B, Nozawa W, Managi S (2020). Sustainability measurements in China and Japan: An application of the inclusive wealth concept from a geographical perspective. Reg Environ Change.

[10] Wujarso R (2012). The Influence of Human Capital on Economic Growth. IJSOC.

[11] Schultz TW (1961). Investment in human capital. AER.

[12] Mincer J. Studies in human capital (Vol. 1). Brookfield, Vermont: Edward Elgar Publishing; 1993.

[13] Fleisher B, Li H, Zhao MQ (2010). Human capital, economic growth, and regional inequality in China. J Dev Econ.

[14] Galor O, Moav O. (2004). From physical to human capital accumulation: Inequality and the process of development. Rev. Econ. Stud. 2004;71(4):1001–.1026.

[15] Becker G (1994). Human Capital: A theoretical and empirical analysis with special reference to Education.

[16] Becker GS, Murphy KM, Tamura R. Human capital, fertility, and economic growth. J. Polit. Econ 1990;98(5, Part 2):S12–S37.

[17] Mincer J (1974). Schooling, Experience and Earning.

[18] Lim SS, Updike RL, Kaldjian AS (2018). Measuring human capital: A systematic analysis of 195 countries and territories, 1990–2016. Lancet.

[19] Hanushek EA, Woessmann L (2008). The role of cognitive skills in economic development. J Econ Lit.

[20] Black SE, Lynch LM (1996). Human-capital investments and productivity. AER.

[21] Schnall PL, Landsbergis PA, Baker D (1994). Job strain and cardiovascular disease. Annu Rev Public Health.

[22] Bakker AB, Demerouti E (2007). The Job Demands-Resources model: State of the art. J Manag Psychol.

[23] Bhui, KS, Dinos, S, Stansfeld, SA, White, PD. A synthesis of the evidence for managing stress at work: a review of the reviews reporting on anxiety, depression, and absenteeism. J. Environ. Public Health. 2012, 515–874. 10.1155/2012/515874 10.1155/2012/515874 PMC3306941 22496705

[24] Aronsson G, Theorell T, Grape T, Hammarström A, Hogstedt C, Marteinsdottir I, Skoog I, Träskman-Bendz L, Hall C (2017). A systematic review including meta-analysis of work environment and burnout symptoms. BMC Public Health.

[25] Einarsen S, Nielsen MB (2015). Workplace bullying as an antecedent of mental health problems: a five-year prospective and representative study. Int Arch Occup Environ Health.

[26] Bonde JPE (2008). Psychosocial factors at work and risk of depression: a systematic review of the epidemiological evidence. Occup Environ Med.

[27] Ganster DC, Rosen CC (2013). Work stress and employee health: a multidisciplinary review. J Manag.

[28] Finne LB, Knardahl S, Lau B (2011). Workplace bullying and mental distress—a prospective study of Norwegian employees. Scand J Work Environ Health.

[29] Hassard, J, Teoh, KRH., Visockaite, G, Dewe, P, Cox, T. The cost of work-related stress to society: a systematic review. J. Occup. Health Psychol. 2018;23:1–17. 10.1037/ocp0000069 10.1037/ocp0000069 28358567

[30] Harvey SB, Modini M, Joyce S, Milligan-Saville JS, Tan L, Mykletun A, Bryant RA, Christensen H, Mitchell PB (2017). Can work make you mentally ill? A systematic meta-review of work-related risk factors for common mental health problems. Occup Environ Med.

[31] Moen P, Kelly EL, Fan W, Lee SR, Almeida D, Kossek EE, Buxton OM (2016). Does a flexibility/support organizational initiative improve high-tech employees’ well-being? Evidence from the work, family, and health network. Am Social Rev.

[32] Kouvonen A, Oksanen T, Vahtera J, Stafford M, Wilkinson R, Schneider J, Väänänen A, Virtanen M, Cox SJ, Pentti J, Elovainio M, Kivimäki M (2018). Low workplace social capital as a predictor of depression: the Finnish Public Sector Study. Am J Epidemiol.

[33] Oksanen T, Kouvonen A, Kivimäki M, Pentti J, Virtanen M, Linna A, Vahtera J (2008). Social capital at work as a predictor of employee health: multilevel evidence from work units in Finland. Soc Sci Med.

[34] Pohling R, Buruck G, Jungbauer KL, Leiter MP (2016). Work-related factors of presenteeism: the mediating role of mental and physical health. J Occup Health Psychol.

[35] Rugulies, R, Aust, B, Madsen, IE. Effort–reward imbalance at work and risk of depressive disorders. A systematic review and meta-analysis of prospective cohort studies. Scand. J. Work Environ. Health. 2017;43:294–306. 10.5271/sjweh.3632 10.5271/sjweh.3632 28306759

[36] Nieuwenhuijsen K, Bruinvels D, Frings-Dresen M (2010). Psychosocial work environment and stress-related disorders, a systematic review. Occup Med.

[37] Stansfeld SA, Shipley MJ, Head J, Fuhrer R (2012). Repeated job strain and the risk of depression: longitudinal analyses from the Whitehall II study. Am J Public Health.

[38] Stansfeld S, Candy B (2006). Psychosocial work environment and mental health—a meta-analytic review. Scand J Work Environ Health.

[39] Too LS, Leach L, Butterworth P (2020). Is the association between poor job control and common mental disorder explained by general perceptions of control? Findings from an Australian longitudinal cohort. Scand J Work Environ Health.

[40] Yang, X, Ge, C, Hu, B. Relationship between quality of life and occupational stress among teachers. Public Health. 20090;123:750–5. 10.1016/j.puhe.2009.09.018 10.1016/j.puhe.2009.09.018 19883926

[41] Umene-Nakano W, Kato TA, Kikuchi S, Tateno M, Fujisawa D, Hoshuyama T, Nakamura J (2013). Nationwide survey of work environment, work–life balance and burnout among psychiatrists in Japan. PLoS ONE.

[42] Chirico F, Capitanelli I, Bollo M, Ferrari G, Acquadro MD (2021). Association between workplace violence and burnout syndrome among schoolteachers: A systematic review. J Health Soc Sci.

[43] Magnavita N, Heponiemi T, Chirico F (2020). Workplace Violence Is Associated With Impaired Work Functioning in Nurses: An Italian Cross-Sectional Study. J Nurs Scholarsh.

[44] Chirico F (2017). Is burnout a syndrome or an occupational disease?. Instructions for occupational physicians Epidemiol Prev.

[45] Chirico F. The assessment of psychosocial risk: only “work-related stress” or something else? Med Lav. 2015;106(1):65–6. 25607288

[46] World health organization. Global burden of mental disorders and the need for a comprehensive, coordinated response from health and social sectors at the country level. 2011. https://apps.who.int/gb/ebwha/pdf_files/EB130/B130_9-en.pdf. Accessed 26 July 2021.

[47] Imtiaz S, Ahmad S (2009). Impact of stress on employee productivity, performance and turnover; an important managerial issue. Int Rev Bus Res Papers.

[48] Kazmi R, Amjad S, Khan D. Occupational stress and its effect on job performance. A case study of medical house officers of district Abbottabad. JMAC 2008;20(3):135–139. 19610539

[49] Muraale S, Basit A, Hassan Z (2017). Impact of job stress on employee performance. Int J Account Bus Manag.

[50] Chirico F (2017). May the gross domestic product growth be a valid indicator of decent work?. Ann Ig.

[51] Ministry of Health, Labour and Welfare. 2021. Accessed from: https://www.mhlw.go.jp/bunya/roudoukijun/anzeneisei12/, Accessed December 21th 2021.

[52] Pew Research Center. Coverage in internet surveys: who web-only surveys miss and how that affects results. 2015. http://www.pewresearch.org/2015/09/22/coverage-error-in-internet-surveys/, Accessed 11 Sept 2020.

[53] Chapman A, Fujii H, Managi S (2019). Multinational life satisfaction, perceived inequality, and energy affordability. Nat Sustain.

[54] Kawakami N, Tsutsumi A (2016). The Stress Check Program: a new national policy for monitoring and screening psychosocial stress in the workplace in Japan. J Occup Health.

[55] Tsutsumi A, Shimazu A, Eguchi H, Inoue A, Kawakami N (2018). A Japanese Stress Check Program screening tool predicts employee long-term sickness absence: a prospective study. J Occup Health.

[56] Fan J, Gijbels I. Local polynomial modelling and its applications: monographs on statistics and applied probability. Boca Raton, Florida: CRC Press; 1996. Vol 66.

[57] Wooldridge J (2010). Econometric Analysis of Cross Section and Panel Data.

[58] Bank of Japan. Average interest rates posted at financial institutions by type of deposit. 2020. https://www.boj.or.jp/statistics/dl/depo/tento/te200813.pdf. Accessed 16 Aug 2020.

[59] Morningstar. Japan US exchange rate. 2020. https://www.morningstar.co.jp/, Accessed 11 Sep 2020.

[60] Gutierrez RG, Linhart JM, Pitblado JS (2003). From the help desk: Local polynomial regression and Stata plugins. Stata J.

[61] Robinson PM (1988). Root-N-consistent semiparametric regression. Econometrica.

