# Supplementary material for: Evaluation of employee occupational stress by estimating the loss of human capital in Japan
Source: BMC Public Health. 2022 Mar 1;22:411. doi: 10.1186/s12889-022-12751-7 (PMC8887011; doi:10.1186/s12889-022-12751-7)
Supplement: Supplementary file 1 — Additional file 1. [file 12889_2022_12751_MOESM1_ESM.docx]

Appendix

Table A1. Men-employee yearly human capital loss at each stress level ranged between 29 to 116

| Reversed GHQ-12 | Job stress-29 | Yearly human capital loss by stress (100 USD) |
| --- | --- | --- |
| 48.00 | 116 | 139.135 |
| 47.59 | 115 | 142.616 |
| 47.17 | 114 | 144.330 |
| 46.76 | 113 | 145.794 |
| 46.34 | 112 | 146.894 |
| 45.93 | 111 | 147.897 |
| 45.52 | 110 | 149.753 |
| 45.10 | 109 | 152.212 |
| 44.69 | 108 | 154.783 |
| 44.28 | 107 | 158.623 |
| 43.86 | 106 | 162.443 |
| 43.45 | 105 | 166.918 |
| 43.03 | 104 | 170.293 |
| 42.62 | 103 | 172.394 |
| 42.21 | 102 | 174.081 |
| 41.79 | 101 | 174.680 |
| 41.38 | 100 | 175.933 |
| 40.97 | 99 | 177.059 |
| 40.55 | 98 | 177.939 |
| 40.14 | 97 | 178.282 |
| 39.72 | 96 | 177.917 |
| 39.31 | 95 | 178.425 |
| 38.90 | 94 | 180.120 |
| 38.48 | 93 | 179.736 |
| 38.07 | 92 | 178.021 |
| 37.66 | 91 | 175.489 |
| 37.24 | 90 | 171.066 |
| 36.83 | 89 | 167.172 |
| 36.41 | 88 | 162.309 |
| 36.00 | 87 | 158.262 |
| 35.59 | 86 | 154.003 |
| 35.17 | 85 | 149.066 |
| 34.76 | 84 | 145.249 |
| 34.34 | 83 | 140.830 |
| 33.93 | 82 | 137.398 |
| 33.52 | 81 | 134.087 |
| 33.10 | 80 | 130.968 |
| 32.69 | 79 | 128.194 |
| 32.28 | 78 | 125.353 |
| 31.86 | 77 | 123.264 |
| 31.45 | 76 | 121.305 |
| 31.03 | 75 | 119.934 |
| 30.62 | 74 | 118.768 |
| 30.21 | 73 | 117.303 |
| 29.79 | 72 | 116.928 |
| 29.38 | 71 | 115.652 |
| 28.97 | 70 | 114.588 |
| 28.55 | 69 | 113.765 |
| 28.14 | 68 | 113.122 |
| 27.72 | 67 | 112.531 |
| 27.31 | 66 | 111.586 |
| 26.90 | 65 | 110.989 |
| 26.48 | 64 | 110.156 |
| 26.07 | 63 | 109.192 |
| 25.66 | 62 | 108.147 |
| 25.24 | 61 | 106.738 |
| 24.83 | 60 | 105.577 |
| 24.41 | 59 | 104.362 |
| 24.00 | 58 | 103.301 |
| 23.59 | 57 | 102.107 |
| 23.17 | 56 | 100.623 |
| 22.76 | 55 | 99.423 |
| 22.34 | 54 | 97.641 |
| 21.93 | 53 | 96.252 |
| 21.52 | 52 | 94.953 |
| 21.10 | 51 | 93.641 |
| 20.69 | 50 | 92.296 |
| 20.28 | 49 | 90.527 |
| 19.86 | 48 | 88.541 |
| 19.45 | 47 | 86.149 |
| 19.03 | 46 | 84.392 |
| 18.62 | 45 | 83.255 |
| 18.21 | 44 | 81.518 |
| 17.79 | 43 | 80.270 |
| 17.38 | 42 | 78.664 |
| 16.97 | 41 | 76.985 |
| 16.55 | 40 | 74.872 |
| 16.14 | 39 | 71.994 |
| 15.72 | 38 | 68.604 |
| 15.31 | 37 | 63.609 |
| 14.90 | 36 | 59.612 |
| 14.48 | 35 | 53.917 |
| 14.07 | 34 | 46.408 |
| 13.66 | 33 | 38.121 |
| 13.24 | 32 | 22.344 |
| 12.83 | 31 | 15.674 |
| 12.41 | 30 | 5.782 |
| 12.00 | 29 | 0.000 |

Note: Data sources: Original global survey. The results are derived from Eq. (1).

Table A2. Macro-approach Men-employee lifetime human capital loss at each stress level

Note: The lifetime human capital loss is calculated by Eq. (4). Data sources: original internet survey

Table A3. Micro-approach Men-employee lifetime human capital loss at each stress level (Eq.3)

Note: The lifetime human capital loss is calculated by Eq. (3).

Data sources: original internet survey


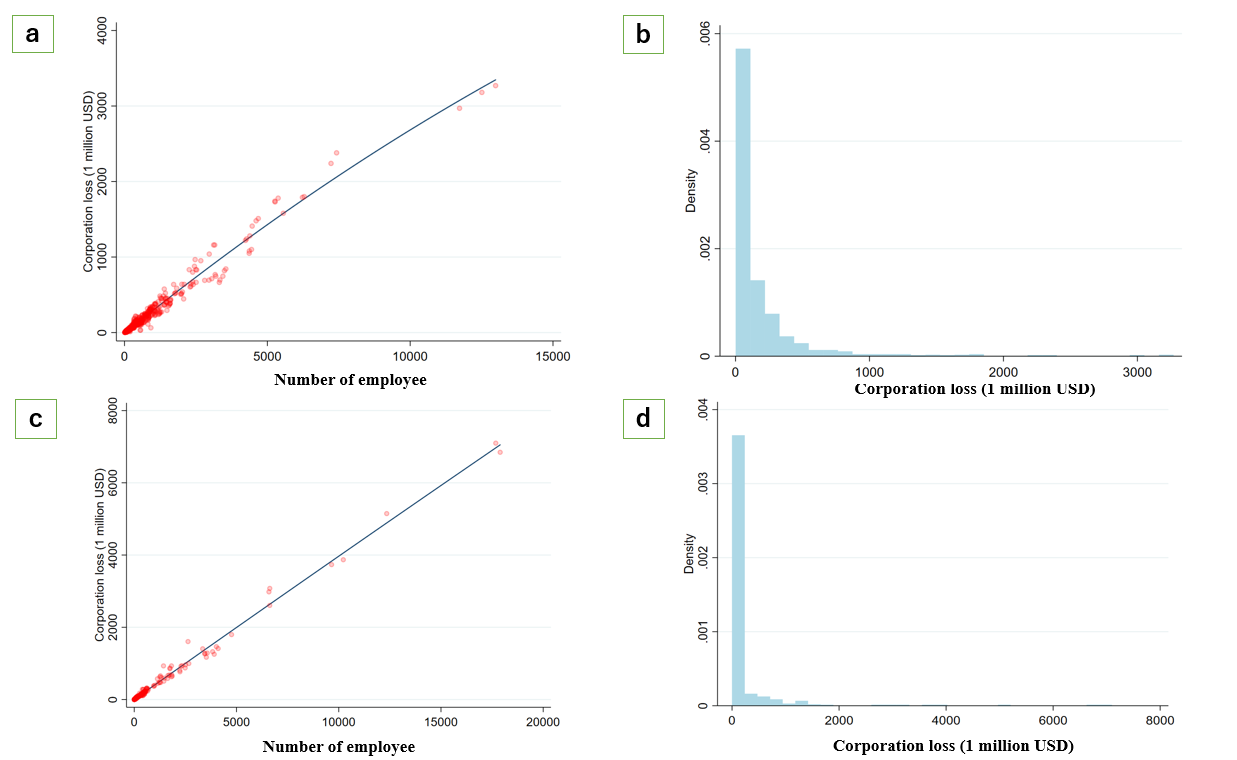


Figure A1. Robustness check of the human capital loss.

Note: The figure (a) and (b), the lifetime human capital loss by stress are derived from micro-approach Eq. (3) with a third-party company. The figure (c) and (d), the macro-approach lifetime human capital and a fourth-party company data is used.


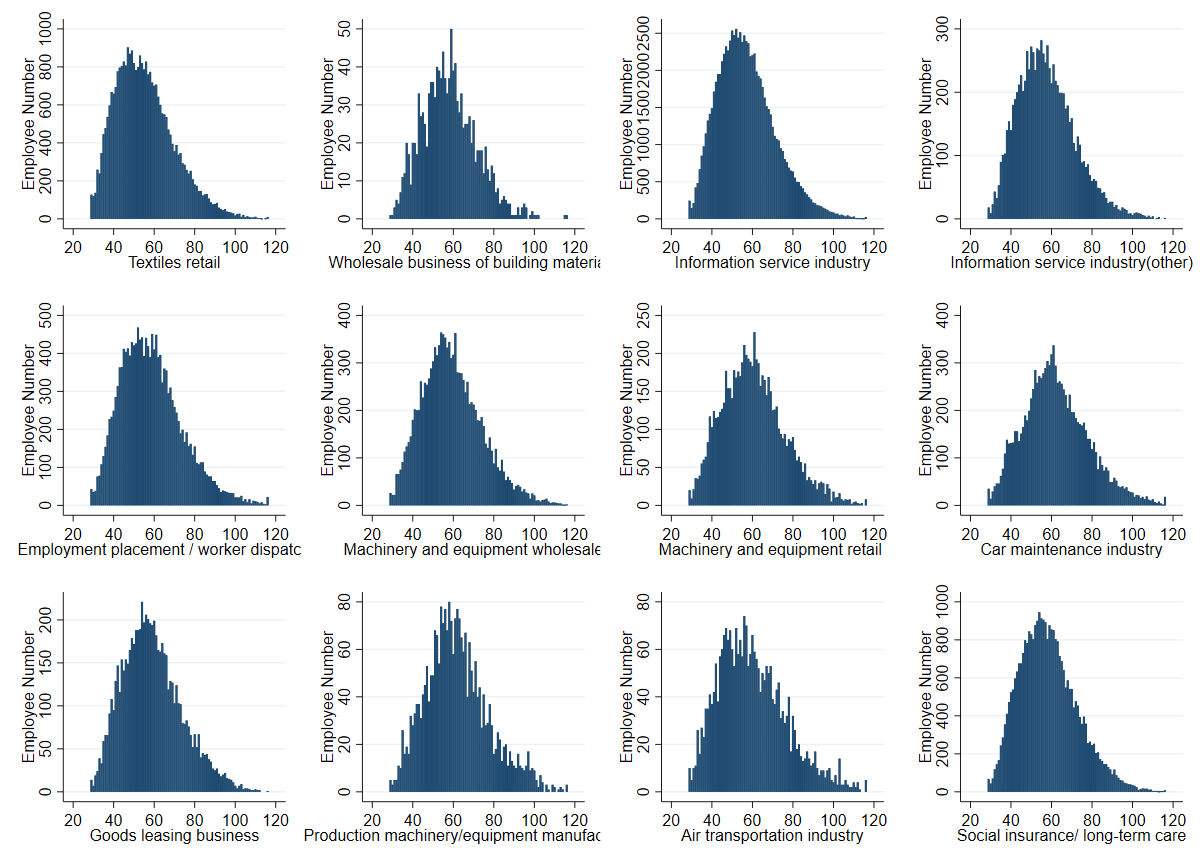


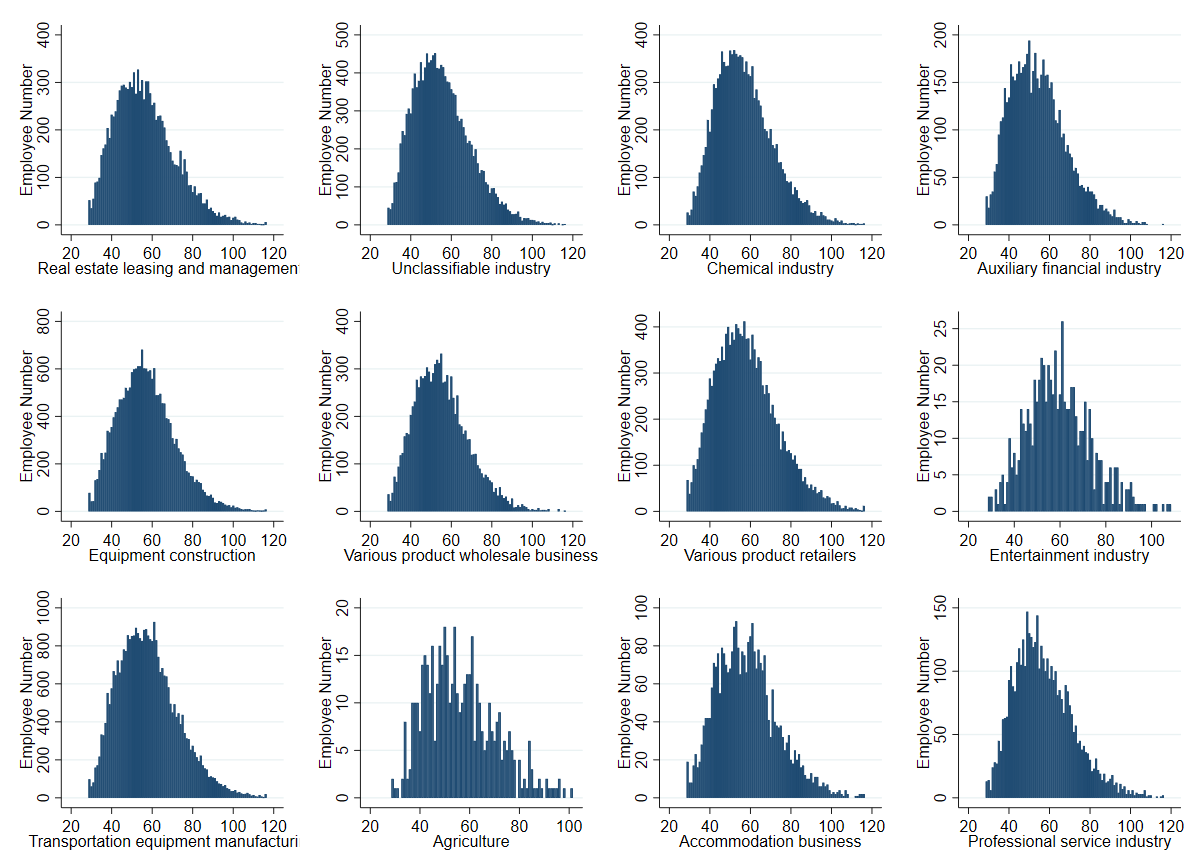


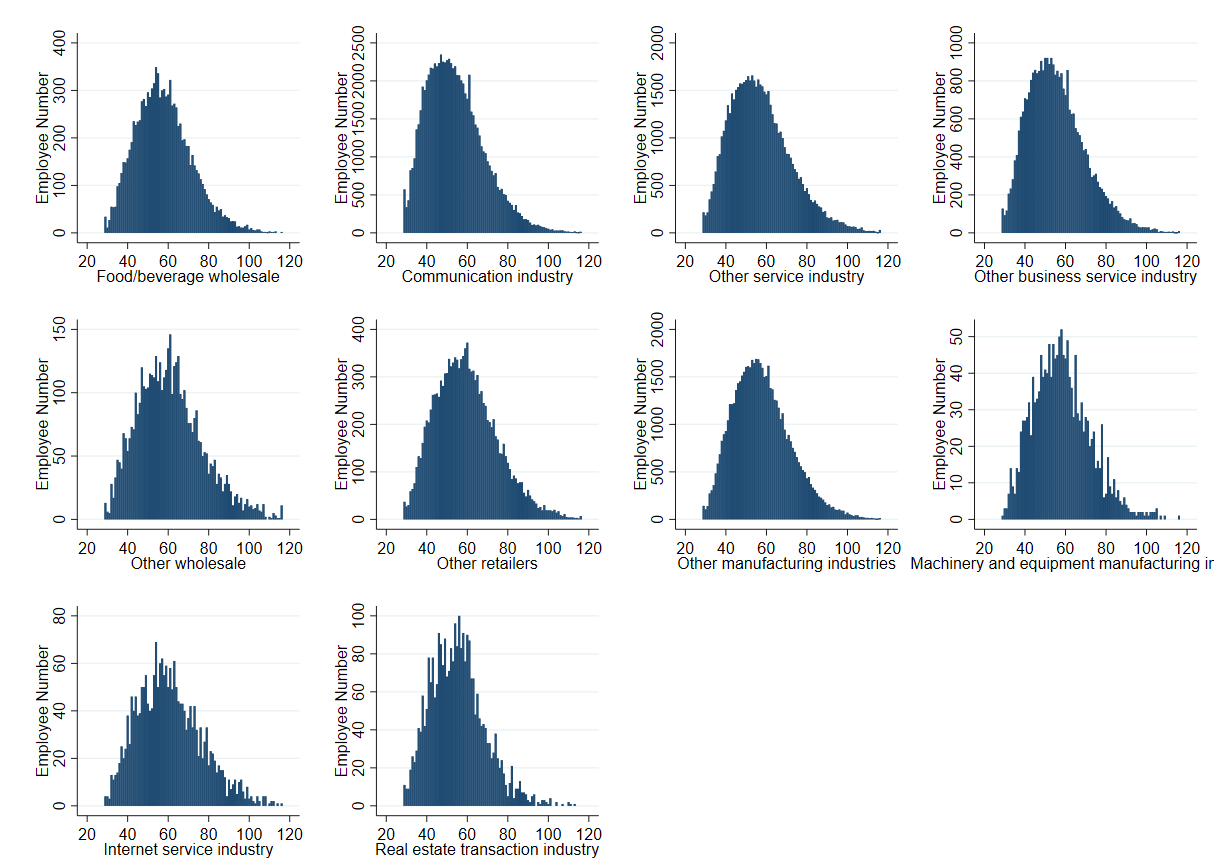


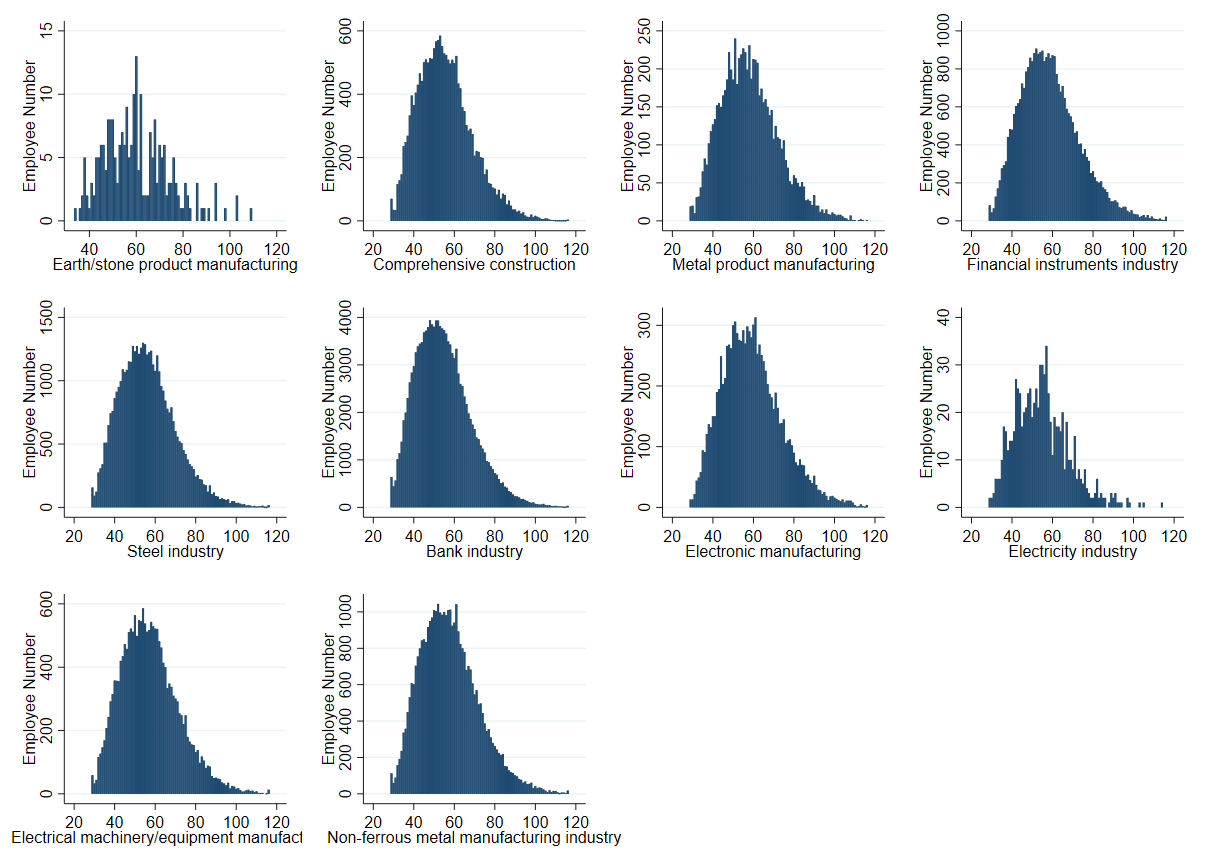


Figure A2. The distribution of employee stress by detailed industry

Table A4. The questionnaire of employee stress conducted by companies.

year Freq. Percent Cum.

2017 371 32.26 32.26

2018 389 33.83 66.09

2019 390 33.91 100.00

Total 1,150 100.00

Table A5 Estimation results of wife's income share 2015.

|  | Wife's income share | | |
| --- | --- | --- | --- |
|  | Coeff. |  | (S.E.) |
| Husband's education attainment |  |  |  |
| (ref. Junior school or lower) |  |  |  |
| Senior high school | -0.070 | *** | 0.020 |
| Vocational school | -0.074 | *** | 0.022 |
| College | -0.055 | * | 0.030 |
| University | -0.083 | *** | 0.022 |
| Graduate | -0.120 | *** | 0.032 |
| Wife's education attainment |  |  |  |
| (ref. Junior school or lower) |  |  |  |
| Senior high school | 0.049 |  | 0.031 |
| Vocational school | 0.082 | ** | 0.032 |
| College | 0.075 | ** | 0.033 |
| University | 0.073 | ** | 0.033 |
| Graduate | 0.096 | * | 0.053 |
| Husband work status (ref. irregular worker) |  |  |  |
| Regular | -0.140 | *** | 0.033 |
| Other | -0.102 | *** | 0.034 |
| Wife work status (ref. irregular worker) |  |  |  |
| Regular worker | 0.235 | *** | 0.011 |
| Other worker | 0.080 | *** | 0.013 |
| Mental health score | 0.001 |  | 0.001 |
| Constant | 0.290 | *** | 0.061 |
|  |  |  |  |
| Observations | 748 |  |  |
| R-squared | 0.448 |  |  |

Note: *** p<0.01, ** p<0.05, * p<0.1.

Data sources: JPSC 2015. Data from the Japanese Panel Survey of Consumers (JPSC), which was conducted by the Institute for Research on Household Economics. The data was collected annually from 1993 until 2017 and includes detailed demographic, income management, income, savings, and expenditures information. The advantage of the data from JPSC is that the detailed household information is collected annually over multiple years with a low rate of dropout households. The first cohort (Cohort A) was recruited nationally in 1993 by randomly selecting young women aged 24 to 34; and new cohorts have been added in 1997 for young women aged 24 to 27 (Cohort B); in 2003, young women aged 24 to 27 were added as Cohort C; in 2008, women aged 24 to 28 were added as Cohort D; finally, in 2013, Cohort E added young women aged 24 to 28. The sample used in this study includes married women and men from 5 cohorts in 2015, and the observations with missing values are deleted.
